# Oral cancer: Current role of radiotherapy and chemotherapy

**DOI:** 10.4317/medoral.18772

**Published:** 2013-02-05

**Authors:** Shao H. Huang, Brian O´Sullivan

**Affiliations:** 1 MD, MRT(T), Assistant Professor; 2 MD, Professor Department of Radiation Oncology, the Princess Margaret Hospital, University of Toronto; 3 ….

## Abstract

The term oral cavity cancer (OSCC) constitutes cancers of the mucosal surfaces of the lips, floor of mouth, oral tongue, buccal mucosa, lower and upper gingiva, hard palate and retromolar trigone. Treatment approaches for OSCC include single management with surgery, radiotherapy [external beam radiotherapy (EBRT) and/or brachytherapy], as well as adjuvant systemic therapy (chemotherapy and/or target agents); various combinations of these modalities may also be used depending on the disease presentation and pathological findings. The selection of sole or combined modality is based on various considerations that include disease control probability, the anticipated functional and cosmetic outcomes, tumor resectability, patient general condition, and availability of resources and expertise. For resectable OSCC, the mainstay of treatment is surgery, though same practitioners may advocate for the use of radiotherapy alone in selected “early” disease presentations or combined with chemotherapy in more locally advanced stage disease. In general, the latter is more commonly reserved for cases where surgery may be problematic. Thus, primary radiotherapy ± chemotherapy is usually reserved for patients unable to tolerate or who are otherwise unsuited for surgery. On the other hand, brachytherapy may be considered as a sole modality for early small primary tumor. It also has a role as an adjuvant to surgery in the setting of inadequate pathologically assessed resection margins, as does postoperative external beam radiotherapy ± chemotherapy, which is usually reserved for those with unfavorable pathological features. Brachytherapy can also be especially useful in the re-irradiation setting for persistent or recurrent disease or for a second primary arising within a previous radiation field. Biological agents targeting the epithelial growth factor receptor (EGFR) have emerged as a potential moda-lity in combination with radiotherapy or chemoradiotherpy and are currently under evaluation in clinical trials.

** Key words:**Radiotherapy, chemoradiotherapy, oral cavity cancer, treatment.

## Introduction

In epidemiology studies, the term ‘oral cancer’ is sometimes employed to connote both oral cavity cancer and oropharyngeal cancer. However, these are different clinical entities and in contemporary practice often have different etiologies and are frequently managed differently. The latter largely occurs for reasons that pertain to local anatomy, functional outcome, and long-term toxicity, especially bone. This review will embrace the clinical definition of “oral cancer”, as defined by the American Joint Committee on Cancer (AJCC) and the Union for International Cancer Control (UICC) in the tumor-node-metastasis (TNM) staging classification. This includes squamous cell carcinomas of the oral cavity (OSCC) originating from the mucosal lip, anterior two-thirds of the tongue (oral tongue), buccal mucosa, floor of mouth, hard palate, lower and upper alveolus and gingiva, and the retromolar trigone.

Treatment of OSCC includes single modality surgery, radiotherapy [external beam radiotherapy (EBRT) and/or brachytherapy], or various combinations of these modalities with or without systemic therapy (chemotherapy and/or target agents). The selection of treatment is based on considerations of disease control, anticipated functional and cosmetic outcomes, and availability of resources and expertise. From a practical stand-point, the availability of reports largely dictates that we need to rely on retrospective case series and a few available randomized trials and several combined analysis that include meta-analysis data.

The mainstay of treatment for OSCC is usually surgery (1). EBRT with or without chemotherapy is generally employed in 3 situations: a) adjuvant to primary surgery to enhance loco-regional control (LRC) for cases with unfavorable pathological features, b) primary treatment for cases unable to tolerate or unsuited for surgery, and c) salvage treatment in the persistent or recurrent disease setting. Brachytherapy may be employed as a sole modality for early disease with a well-defined primary tumor, or as an adjuvant to surgery for cases with close or positive resection margins. Alternatively it may be used as a “boost” technique to the primary tumor in addition to EBRT ( Table 1). Recently, epithelial grown factor receptor (EGFR) targeted therapy, such as cetuximab, has emerged as a promising treatment option in conjunction with EBRT to enhance disease control.

**Table 1:**
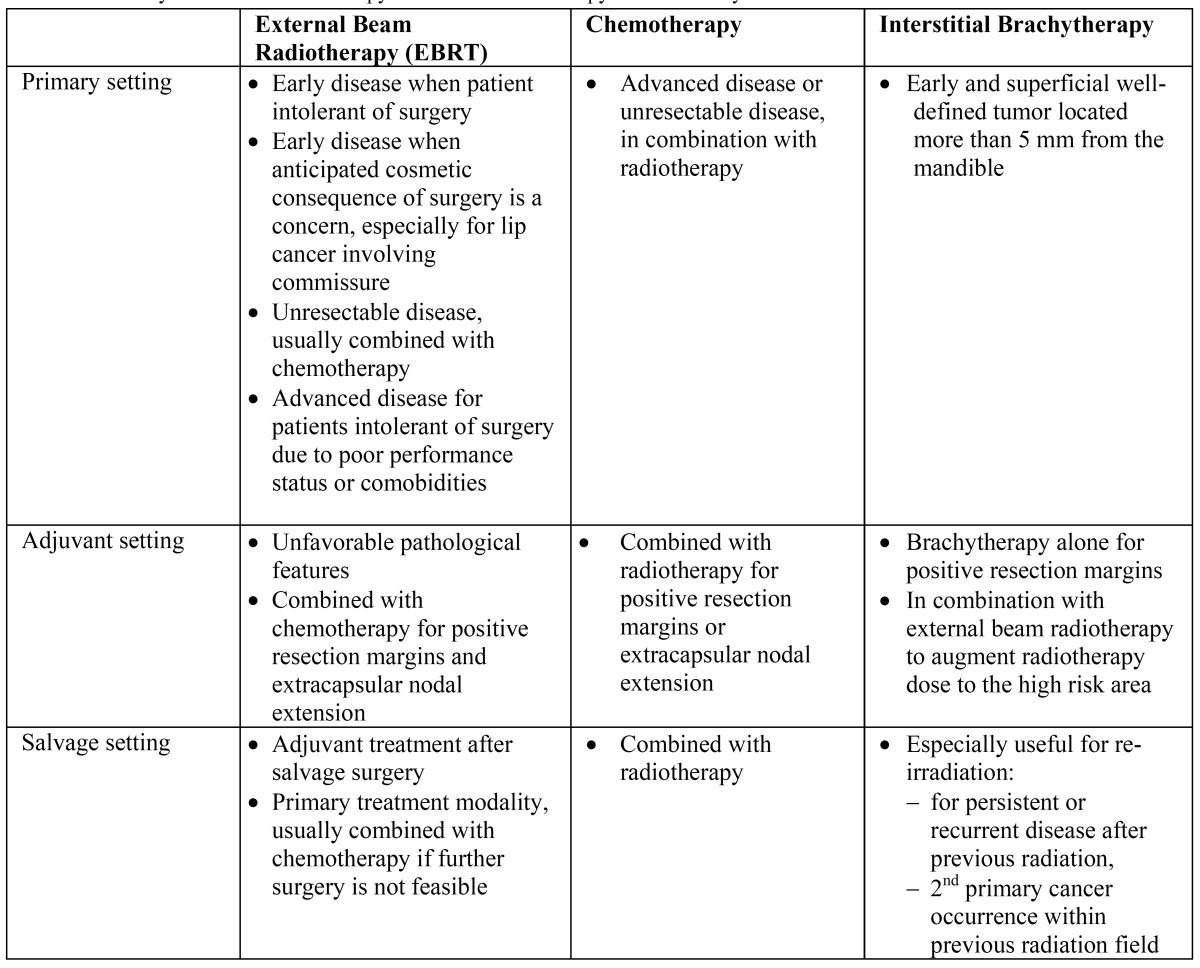
Summary of Role of Radiotherapy and Chemoradiotherapy in Oral Cavity Cancer.

## Postoperative Radiotherapy ± Chemotherapy

-Risk profile and indications for adjuvant treatment

Primary surgery is the traditional approach for resectable OSCC in most centers. For patients with unfavourable pathological features, postoperative radiotherapy (PORT) or postoperative concurrent chemo-radiotherapy (POCRT) have been shown to improve LRC and survival in several clinical trials (2-4). General indications for PORT include: T3 or T4 tumor; compromised surgical resection margins (<5 mm from the inked surface of the specimen); presence of lympho-vascular invasion (LVI) and/or peri-neural invasion (PNI); and positive lymph nodes with or without extracapsular invasion (ECE) (2,5).

The presence of multiple risk factors is common in OSCC. To understand the loco-regional recurrence risk profile, Langendijk, et al. studied 801 head-and-neck cancer patients (73% of whom had OSCC) who underwent PORT in 1985-2000. They did not include their most favourable cases that did not require PORT in the report. A recursive partitioning analysis addressing loco-regional recurrence stratified patients into three risk groups: a) intermediate-risk: clear resection margins and no ECE, b) high-risk: T1, T2 and T4 tumor with close or positive margins or one pathological positive lymph node with ECE, c) very high-risk: T3 tumor with close or positive surgical margins or multiple pathological positive lymph nodes with ECE or an N3 neck (6). For the latter two groups, the 5-year LRC with PORT was unsatisfactory (78% and 58%, respectively). The authors concluded that more intensive approaches, such as POCRT with concurrent chemotherapy should be considered for these two sub-groups.

Pathological stage I-II disease with sufficiently clear resection margins is generally considered low-risk and does not require PORT (7). Studies suggest that PNI alone appears to be non-predictive for recurrence (8,9). However, the presence of LVI or microscopic tumor foci in muscle increased the risk of recurrence and PORT should be considered. Tumor thickness, or alternative synonyms such as “depth of invasion” or “tumor depth”, has been consistently identified as a predictor for cervical lymph node metastasis (10). Recent studies have shown that pathological tumor thickness ≥ 4 mm combined with poorly differentiated pT1-2N0 OSCC tumors are associated with poor regional control and such patients may benefit from PORT (11).

The effectiveness of PORT for T1 or T2 primary with completely resected N1 disease is yet to be confirmed. Currently, there is an ongoing European clinical trial evaluating the effectiveness of PORT in pT1-T2pN1 oral cavity and oropharyngeal cancers with clear resection margins (12).

**Figure 1:**
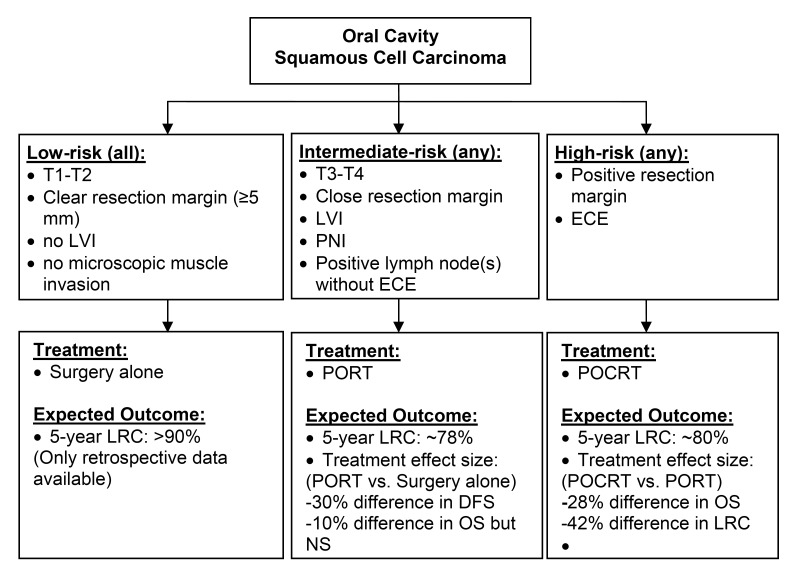
Summary of Risk Grouping and Role of Postoperative Radiotherapy +/- Chemotherapy. Abbreviations: LVI: lympho-vascular invasion; ECE: extra-capsular invasion; PORT: postoperative radiotherapy; POCRT: postoperative chemoradiotherapy; LRC: locoregional control; DFS: disease-free survival; OS: overall survival; NS: not statistical significant Note: 1. Crude estimates of expected outcomes were obtained from the following sources: • Low-risk: Huang, et al. (8) • Intermediate-risk: Langendjk, et al. (25), • High-risk: Bernier, et al. (2) from the experimental arms of the combined report of RTOG #9501 and EORTC #22931 2. Treatment effect size: • PORT vs. Surgery: Mishra, et al (55) • POCRT vs. PORT: Bernier, et al (2)

The presence of microscopic positive margins and/or ECE is considered high-risk for recurrence. The addition of chemotherapy to PORT for these patients resulted in a 13% absolute reduction in loco-regional relapse at 5-years in the EORTC trial 22931 reported by Bernier et al. (13) and a 10% absolute reduction at 2-years in the RTOG trial 9501 reported by Cooper, et al. (3). Other features may also be considered “high-risk”. The RTOG 85-03 and 88-24 trials indicated the presence of 2 or more pathological positive lymph nodes as a high-risk feature for loco-regional failure (14). Some studies have also shown that multiple (> 2) minor risk factors combined with LVI were associated with poor prognosis (15-17) suggesting that more intensive regimens such as concurrent chemotherapy, may be justified in addition to PORT (Fig. 1).

-Definition of close resection margin

The definition of ‘close’ resection margins is generally accepted as tumor within 5 mm of the inked resection margin in formalin fixed surgical specimens (18). Several small retrospective studies suggested that the traditional margin of 5 mm may be too generous, especially when one consider the shrinkage that take place in the final surgical specimen; this may amount to as much as 40%~50% compared to fresh specimens (19). These authors suggested that a 2 mm inked margin as “cutoff” for the “close” margin definition is sufficient (20,21). However, these findings are based on small retrospective studies and need confirmation.

The risk associated with an intra-operatively ‘revised’ margin following a positive resection margin excision has not been well studied. Compromised local control and disease-specific survival for those with intra-operative positive margin was described recently even though the final tumor margin was negative after revision (22). The latter study supports the adverse nature of an intra-operative positive margin regardless of final tumor margin, and PORT should be considered.

-Optimal surgery-to-radiotherapy interval

The optimal surgery-to-radiotherapy interval is controversial. A systematic review of published literature showed an increased odds ratio (2.9) for local recurrence in head and neck cancer patients whose PORT were started more than 6 weeks after surgery vs. those within 6 weeks of surgery (23). The optimal nature of a surgery-to-radiotherapy interval of “6 weeks” was also echoed in a recent OSCC series (24) but not confirmed by others (5,25). Nevertheless, commencing PORT as soon as possible seems desirable but requires careful planning and multidisciplinary collaboration, and may not always be achievable when surgical complications are present. Furthermore, such complications are more likely following larger and more extensive surgical resection for more locally invasive lesions, larger tumors, and those with extensive lymph node involvement. Patients with such tumors are more likely to be candidates for intensive adjuvant treatments and the most likely to suffer from their omission. The greater prevalence of more advanced cancer in the latter time cohort due to wound healing delay may also partly explain any putative adverse influence of delay in the initiation of PORT. Therefore, no arbitrary time limit has been scientifically established during which PORT must begin, or beyond which PORT has been shown not to have an effect (5). In essence, high risk cases should still be considered in circumstances where there has been delay in initiating radiotherapy due to the grave consequences of loco-regional recurrence that might be prevented by the use of adjuvant treatment.

## Primary Radiotherapy ± Chemotherapy without Surgery

Primary radiotherapy with or without chemotherapy is not used routinely but may be deployed for the following reasons: a) in early stage disease to avoid anticipated functional and cosmetic defect, b) for unresectable disease, c) for high operative risk patients due to comorbidity or poor performance status, d) recurrent disease when previous multiple surgeries have been undertaken and further surgery would be technically improbable, and e) patient’s preference. No prospective trial has directly compared primary surgery vs. primary radiotherapy in OSCC specifically. Two case series comparing RT vs. surgery suggested a lower LRC with primary radiotherapy compared to surgery approach (26,27). However, these patients were mostly treated with less intensified treatment regimens and the retrospective nature of the studies questions whether case selection for both treatments was equal. Also, whether intensified treatment approaches, such as concurrent chemo-radiotherapy, could improve outcome sufficiently to be comparable to surgery remains to be investigated. A subgroup analysis of 39 T4 OSCC with primary CRT from four multi-institutional phase II studies showed a 5-year LRC rate of 75% (28); in turn this opens the potential for organ preservation approaches with CRT in patients with T4 OSCC.

In the meta-analysis of individual patient data from clinical trials comparing RT vs. CRT (MACH-NC) in locally advanced head and neck cancers, OSCC comprised 21% of cases (29). Result showed an improvement of survival in OSCC with CRT compared to RT alone (HR =0.8). In the meta-analysis of individual patient data from clinical trials comparing hyper-fractionated or accelerated vs. conventional radiotherapy schedule (MARCH) (30), OSCC consisted of only 12.6% of cases. Again, intensified regimens, in this case altered fractionation radiotherapy, improved survival over conventional radiotherapy (hazard ratio = 0.8) . The results from these two meta-analyses seem to suggest that for patient unable to receive primary surgery, treatment intensification by concurrent chemotherapy or altered fractionation RT may be considered.

## Interstitial Radiotherapy (Brachytherapy)

Interstitial brachytherapy represents a traditional approach for OSCC and is an alternative to external beam radiotherapy (EBRT). Brachytherapy delivers radiotherapy by positioning radioactive sources in direct proximity to the tumor target area. The advantage of brachytherapy is its highly conformal dose distribution to a small target area by virtue of a rapid “fall-off” within surrounding normal tissue. It can be applied as a definitive treatment for early OSCC; as a complimentary treatment in combination with surgery; as a local “boost” in combination with EBRT to enhance the local dose to the immediate tumor region; or as a salvage option for small burden persistent or recurrent disease (31).

-Brachytherapy alone

No randomized trials have been performed comparing brachytherapy versus other conventional treatment for OSCC. Based on clinical experience, a consensus statement from GEC-ESTRO has recommended that brachytherapy alone could be used for T1-T2N0 oral mucosa (<1.5 cm in thickness), oral tongue lesion, or floor of mouth lesions locate at least 5 mm from the mandible due to the high risk of inducing osteoradionecrosis (ORN). Brachytherapy is not suitable for T4 tumor with bone involvement (31). An additional concern about the use of brachytherapy alone for early OSCC is its inability to addressing occult neck disease. For example, an increased rate of late lymph node recurrence for clinical T1N0 OSCC has been reported following brachytherapy alone, especially in tumors exceeding 6 mm in thickness. The authors have consequently recommended prophylactic nodal irradiation in addition to brachytherapy for early-stage OSCC exceeding this thickness (32).

-Postoperative brachytherapy

Postoperative brachytherapy could be offered in T1-3 tumor with narrow or positive resection margins or LVI. A retrospective study of local tumor excision followed by postoperative interstitial brachytherapy with and without external radiotherapy has shown excellent loco-regional control (33). In addition, brachytherapy can be delivered as an adjuvant “boost” to augment the radiation dose to a high risk area for advanced OSCC undergoing EBRT (34-37). An alternative approach is the use of simultaneous integrated boost IMRT but the relative value of both approaches remains to be determined.

Brachytherapy for re-irradiation

The feasibility of re-irradiation with EBRT after definitive EBRT is limited by concerns about excessive morbidity related to normal tissue tolerance. Therefore, surgical resection is often and appropriately presented as the main or potentially only curative option. However, carotid involvement in persistent disease is relatively common in recurrent disease and carotid artery resection carries significant risk including potential dramatic adverse sequelae, such as cerebrovascular event. Because of the ability to curtail the size of the irradiated volume, brachytherapy seems especially indicated to minimize the risk of severe complications in re-irradiation for persistent/recurrent disease or for new primary tumors located within previously irradiated volumes (33,38,39).

-Brachytherapy delivery techniques

Commonly used radioactive sources are I-125 and Ir-192. Considerable experience has been accumulated with low-dose rate (LDR) brachytherapy and its effectiveness for OSCC has been confirmed in large clinical series (40). Recently, high-dose rate (HDR) and pulsed-dose rate (PDR) brachytherapy have emerged as new brachytherapy delivery techniques offering the advantage of optimizing dose distribution by varying dwell times and employing computerized planning and delivery techniques. However, these techniques require the availability of particular expertise and resources that has affected their widespread adoption in many centers especially in settings with insufficient operating room resources and radiotherapy protection requirements.

## Biotherapy with Targeted Agent

Emerging data also indicate that the epidermal growth factor (EGFR) and its signal transduction pathway play an important role in head and neck cancer. Over-expression of EGFR has been confirmed in OSCC and has been reported to be associated with a poor prognosis (41-43). The addition of a targeted agent, such as a monoclonal antibody EGFR inhibitor, has been reported to improve outcome over primary radiotherapy alone in head and neck cancers of the oropharynx, hypopharynx and larynx (44). Its role in OSCC has yet to be confirmed. Recently, the RTOG 0920 trial was launched to address intermediate-risk (PNI, LVI, close margin, T3 or T4a tumor, T2 with > 5mm thickness, single lymph node > 3 cm or ≥ 2 lymph node < 6 cm without ECE) OSCC patients to evaluate whether the addition of cetuximab to PORT will improve overall survival (OS) in postoperative patients.

## Radiation-related Toxicity ± Chemotherapy

**Figure 2:**
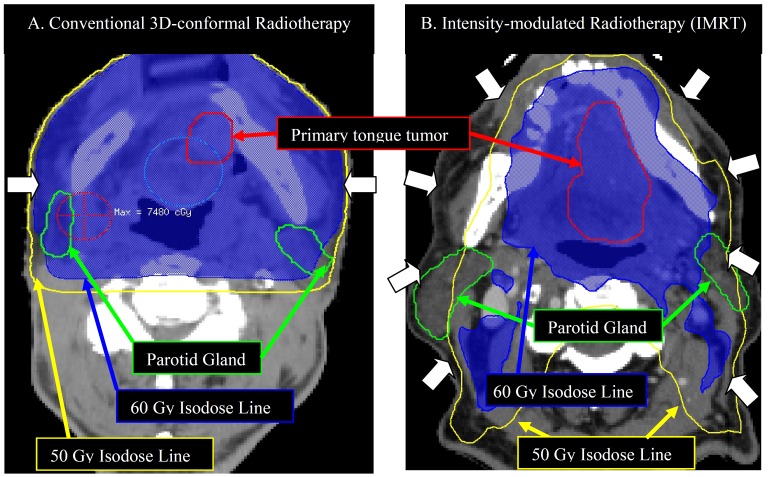
Example of partial mandible and parotid-sparing IMRT vs. conventional 3D-conformal radiotherapy. Note: •Two actual cases of T4aN0 oral tongue cancer. The dose prescription is 70 Gy in 35 fractions in both cases •White arrows indicate radiation beam arrangement: • Conventional radiotherapy: two lateral beams: 90° and 270° • IMRT: equally spaced 9 beam plan configuration: 200°, 240°, 280°, 320°, 0°, 40°, 80°, 120°, 160° •For the same stage of disease, IMRT is able to partially spare the ipsi-lateral and totally spare the contra-lateral mandible and parotid gland to receive 60 Gy; while both side of mandibles and almost both parotid glands received >60 Gy in conventional 3D-conformal radiotherapy.

Radiotherapy with or without chemotherapy to enhance disease control is associated with radiation-induced toxicity. The acute toxicity includes grade 2/3 oral mucositis and dysphagia. The incidence is generally high, especially with bilateral irradiation, though is usually self-limiting (45). Common late toxicities include ORN, xerostomia, and dysphagia (46). The general impression of practitioners is that toxicity is enhanced by the use of concurrent chemotherapy with PORT. For example, the combined severe acute and late toxicity frequency in the RTOG trial 9501 reported by Cooper, et al. (3) amounted to 46% vs. 78% for PORT vs. POCRT respectively. For reasons that are not immediately apparent, the toxicity profile seems less clear in EORTC 22931; for example the severe muscular fibrosis rate was higher for POCRT (10% vs. 5%) but severe xerostomia was lower (14% vs. 22%) (13). These observations underline the need to prospectively include comprehensive toxicity data collection in future trials. Spontaneous ORN is dose-dependent and related to the volume of mandible receiving radiotherapy beyond 50~60 Gy (47). The incidence is generally low (<15%) with conventional conformal radiotherapy (48) with the exception of one unexpected retrospective report in the recent literature (49). Intensity modulated radiotherapy (IMRT) is a newer method of radiotherapy that uses intensity-modulated beams that can provide multiple intensity levels allowing concave dose distributions and dose gradients with narrower margins than those possible using traditional radiotherapy (50) (Fig. 2).

The incidence of ORN can be further reduced significantly by minimizing the percentage of mandibular volume exposed to >50~60 Gy using IMRT (47,51,52). In addition, careful oral dental hygiene and smoking cessation are also important for preventing ORN. Xerostomia rates are high with conventional radiotherapy when salivary gland and oral cavity mucosa are inevitably included in the radiation field. IMRT has demonstrated an ability to enhance salivary sparing to reduce the rate of permanent xerostomia without compromising disease control (45,53). It is also capable of reducing the rate of severe dysphagia compared to conventional radiotherapy (54).
